# Optimizing Intervention Components of a Preventive Stress Management mHealth Intervention for Health Care Workers: Experimental Factorial Study

**DOI:** 10.2196/71032

**Published:** 2025-08-25

**Authors:** Leo Kowalski, Anna Finnes, Sabine Koch, Aleksandra Bujacz

**Affiliations:** 1 Health Informatics Centre, Department of Learning, Informatics, Management, and Ethics, Karolinska Institutet, Tomtebodavägen 18a, Stockholm, 171 65, Sweden, 46 706758656; 2 Division of Psychology, Department of Clinical Neuroscience, Karolinska Institutet, Stockholm, Sweden; 3 Academic Primary Healthcare Centre, Region Stockholm, Stockholm, Sweden

**Keywords:** digital behavior change intervention, mHealth, work stress, factorial experimental design, stress management, mental health, formative assessment, multiphase optimization strategy

## Abstract

**Background:**

Work stress is a prevalent risk factor for mental health problems, such as burnout and depression. Health care workers, especially during the COVID-19 pandemic, face high levels of work stress that make them a vulnerable population in need of support. Digital interventions are a promising way to combat this issue, offering the possibility of scalable programs that are easily accessible. While a wide range of stress management techniques can be incorporated into digital interventions, applying the multiphase optimization strategy enables systematic evaluation of what specific content most contributes to preventing the negative effects of work stress.

**Objective:**

The primary aim of this research was to identify which digital intervention components and combinations of components are most effective at preventing symptoms of stress-related health problems. These insights are valuable to inform future intervention development for optimizing intervention design.

**Methods:**

This study tested 5 digital intervention components aimed at improving stress management among workers. Engagement and Demands components allow participants to self-reflect on their work engagement and work challenges, while the Control component aids a more action-oriented process considering job crafting and detachment strategies. The Journaling component encourages a deeper reflection, and the Psychoeducation component provides evidence-based strategies for managing stress. In an experimental factorial study, Swedish health care workers (n=297) tested different versions of the intervention containing all possible combinations of these 5 components. Stress-related health outcomes, such as burnout, anxiety, and depression, were measured using questionnaires immediately before, immediately after, and 1 month after the end of the intervention.

**Results:**

The most promising intervention effects were observed when the Demands and Control components were present together in the intervention. Including these components led to an increase in social support (β=0.68; *P*<.001) and job crafting (β=0.41; *P*=.06) during the intervention, as well as a decrease in symptoms of emotional exhaustion (β=−0.50; *P*=.005), burnout (β=−0.54; *P*=.004), and anxiety (β=−0.44; *P*=.04) after the intervention. Notably, including one of the components without the other made outcomes worse than including neither of these 2 components. Furthermore, mindfulness after the intervention increased when both the Engagement and Demands components (β=0.72; *P*=.001) were included as well as when the Journaling and Psychoeducation components were included (β=0.46; *P*=.04).

**Conclusions:**

Results indicate that components aiding self-insight should be integrated with components providing actionable advice to achieve optimal intervention effects. Results from this optimization study may inform the development of preventive digital stress management interventions to be tested in future randomized controlled trials.

## Introduction

### Work Stress

Stress has been termed the epidemic of the 21st century, reflecting both its widespread prevalence and adverse health effects [1,2]. Work is a common source of stress, with work-related stress defined as “the harmful physical and emotional responses that occur when the requirements of a job do not match the capabilities, resources, or needs of the worker” by the National Institute for Occupational Safety and Health [3]. This mismatch may have severe implications, as chronic exposure to work stress is a well-known risk factor for mental health problems, such as burnout and depression [4-6].

Alarmingly, work stress is very prevalent: a 2023 national survey by the American Psychological Association indicated that 77% of US workers experienced work stress in the previous month, with 57% reporting negative impacts because of this [7]. Beyond the detrimental effects of work stress on the individual level, stress-related health problems have a substantial economic impact as they are linked with increased sick leave, turnover rates, and productivity loss [8]. A 2024 report by the European Trade Union Institute estimates that costs due to depression attributable to psychosocial work exposures are upwards of US $114 billion in the European Union [9].

Health care workers are particularly vulnerable to work-related stress, with high levels of burnout and stress-related health problems within this population [10]. This issue was further exacerbated during the COVID-19 pandemic when health care workers faced unusually high work demands, such as increased workload and lack of equipment, leading to increases in mental health issues [11,12]. Proactive initiatives that support employees through primary prevention, intervening before work stress leads to health problems, are urgently needed to decrease individual issues and social costs.

### Digital Interventions

Preventive interventions targeting health promotion may improve employee health and well-being, and by extension provide beneficial effects for organizations in terms of increased productivity, job satisfaction, and higher work engagement [13-15]. Digital interventions are among the most promising options for providing accessible and scalable support to workers and may constitute a valuable complement to other efforts. Compared with conventional on-site interventions, such as support groups and counseling, digital tools offer considerable advantages for standardizing interventions [16,17]. These solutions have been found effective for stress-related health outcomes, such as perceived stress, depressive symptoms, and emotional exhaustion within an organizational context [18-20].

Digital Behavior Change Interventions (DBCI) may be well-suited for addressing work stress. These interventions support users in adopting new behaviors and can be effective in a range of behavioral and health outcomes, including physical activity, dietary behavior, and improved mental well-being [21-23]. DBCIs may help workers develop a habit of preventive stress management techniques that mitigate the negative consequences of stress, thereby decreasing the risk of developing stress-related health problems [24,25].

### Intervention Components

#### Overview

To develop a successful DBCI, it is necessary to choose what specific components to include, that is, distinct sets of content or functionality that support intervention aims. Common intervention approaches are often insufficient for determining what set of components provides optimal intervention effects. Complex interventions, for instance, cognitive behavioral therapy (CBT), integrate several components as part of the intervention, making it difficult to tease apart the effect of each individual component [26]. Other approaches, such as Mindfulness-Based Stress Reduction, function primarily through one component (mindfulness) without exploring the benefits of integrating other stress management techniques as part of the intervention [27]. While the following suggested components are evidence-based strategies for stress prevention, it is necessary to investigate the effectiveness of their combinations.

#### Stress Management for Work Stress

As a first step, it is necessary to choose a set of intervention components to consider. Given the intended intervention aims, components should serve to improve stress management for preventing the onset of stress-related health problems. While there is a wide range of evidence-based stress management techniques in academic literature known to alleviate stress reactions and improve mental health (eg, physical activity, mindfulness, and social support), some techniques are especially appropriate in the context of a digital intervention for workers [27-29].

First, it may be important for workers to self-monitor job demands, which are defined as aspects of the job that require sustained physical, cognitive, or emotional effort, to better understand what aspects of the work environment affect their stress levels and health [30]. Job demands are a well-known contributor to work stress, and self-monitoring may be an effective way to increase awareness of these. In addition, interventions targeting work engagement, a positive, energized, and focused state of mind, have been found to be effective in a range of relevant outcomes, including job satisfaction and well-being [15,31]. Combining work engagement elements with awareness of job demands could also be fruitful—this is similar to positive work reflection interventions, which can improve mental health in a working context [32,33].

Another strategy that may be effective is making employees more aware of how to exert control in their lives, both at work and during their free time [34]. Job crafting interventions, that is, programs that help employees shape their work to better fit their needs, are widely considered effective for combating work stress [35]. Likewise, learning to detach from work-related thoughts during leisure time is important for effective recovery [36]. This control component can potentially be combined with raising awareness of job demands. For instance, an intervention teaching job crafting strategies in relation to job demands had positive effects on need satisfaction and work engagement [37].

Journaling, a self-reflection practice in which one expresses thoughts and emotions in writing, is another technique that can have positive effects on mental health for workers. Dimitroff et al [38] found evidence that journaling may reduce burnout and compassion fatigue, while MacIsaac et al [39] suggest that journaling may increase self-reflection, which mediates improved well-being. In addition, incorporating psychoeducation*,* the structured provision of relevant evidence-based information, regarding recovery strategies may be helpful, as these behaviors are known to relieve short-term stress reactions [25,40]. As an example, a behavioral stress recovery intervention indicated a decrease in perceived stress and burnout symptoms [41]. Combining Psychoeducation with a Journaling component is common in therapeutic practices, such as CBT, and may yield a fruitful integration. Asplund et al [42] found positive effects on mental health and well-being from an intervention, including a stress diary and psychoeducation regarding recovery.

#### Multiphase Optimization Strategy

Research is needed to determine what components, Demands, Engagement, Control, Journaling, and Psychoeducation, are most effective in an mHealth intervention for work stress among health care workers. To guide decisions on what specific components to include, the Multiphase Optimization Strategy (MOST) offers a systematic and iterative approach to building and evaluating multicomponent interventions [43]. In the optimization phase of this framework, multiple components and their interactions are tested to better understand their effect on selected outcomes. Factorial experimental designs play a key role in helping to identify which components and combinations of components are most effective. Using these designs, it is possible to test the effect of multiple intervention components in the same trial rather than conducting separate experiments for each component [44].

### Aims and Objectives

By identifying the most effective intervention components for stress management, we may improve digital interventions for work stress and thereby decrease stress-related health problems in the working population. Thus, the aim of the study was to investigate which intervention components and their combinations are most effective in preventing the negative effects of work stress.

1. What single components or specific combinations of components are most effective at preventing symptoms of stress-related health problems?
2. What is the effect of intervention components during as compared to after the intervention?

## Methods

### Participants

This study was conducted as part of a larger research project examining the impact of COVID-19 and psychological support initiatives on Swedish health care staff during the pandemic. The particular organization and timeframe were determined through a research partnership with a local government organization, following the urgent need for developing scalable psychological support solutions during the pandemic.

For recruitment, employees were initially informed by managers about the opportunity to participate and subsequently received an invitation email from the research team. As part of a related data collection in December 2020-January 2021, 1267 health care workers were asked in an electronic survey whether they wanted to take part in this study, to which 297 employees agreed. We used broad inclusion criteria to ensure representation across diverse roles and settings. Participants were eligible if they were currently employed in any capacity within the health care sector. Table 1 shows demographic characteristics of all participants.

**Table 1.** Participant demographics of Swedish health care workers immediately before a 1-month intervention (Preintervention), immediately after the intervention (Postintervention), and 1 month after the end of the intervention (Follow-up).^a^ Occupation category “Other” included, for example, ambulance drivers, technicians, counselors, and physiotherapists. Burnout was measured by the Oldenburg Burnout Inventory. Depression was measured by the Patient Health Questionnaire. Posttraumatic stress disorder was measured by selected items from the Posttraumatic Stress Disorder Checklist. Anxiety was measured by Generalized Anxiety Disorder. Stress was measured by the Perceived Stress Scale. Mindfulness was measured by the Mindful Attention Awareness Scale. Job crafting was measured by Recovery Experience Questionnaire and Job Crafting Questionnaire. Social support was measured by the Job Crafting Questionnaire. Recovery was measured by the Recovery Experience Questionnaire. Emotional exhaustion was measured by the Shirom-Melamed Burnout Questionnaire.

Group	Preintervention measure (n=283)	Postintervention measure (n=218)	Follow-up measure (n=189)
Gender, n (%)			
Women	230 (82.4)	176 (81.1)	154 (81.9)
Men	49 (17.6)	41 (18.9)	34 (18.1)
Occupation, n (%)			
Assistant nurse	16 (5.7)	11 (5.1)	9 (4.8)
Nurse	74 (26.4)	47 (21.8)	43 (22.9)
Doctor	46 (16.4)	45 (20.8)	37 (19.7)
Admin	50 (17.9)	38 (17.6)	33 (17.6)
Other	94 (33.6)	75 (34.7)	66 (35.2)
Tenure (years)			
0‐1	8 (3.0)	6 (2.9)	2 (1.1)
2‐5	62 (23.0)	41 (19.6)	35 (19.3)
6‐10	47 (17.4)	36 (17.2)	30 (16.7)
more than 11	153 (56.6)	126 (60.3)	114 (63.0)
Age (years), mean (SD)	45.4 (12.1)	46.8 (12.0)	47.3 (11.7)
Mental health (score range), n (%)			
Burnout (1-4)	2.42 (0.68)	2.36 (0.68)	2.25 (0.68)
Depression (1-4)	1.72 (0.74)	1.69 (0.73)	1.59 (0.70)
PTSD^b^ (0‐4)	2.06 (0.93)	1.97 (0.89)	1.79 (0.80)
Anxiety (1-4)	1.67 (0.61)	1.63 (0.55)	1.50 (0.48)
Stress (1-5)	3.00 (0.35)	2.99 (0.36)	2.94 (0.34)
Mindfulness (1-6)	2.97 (1.25)	3.11 (1.19)	3.00 (1.126)
Job crafting (1-7)	4.68 (0.91)	4.51 (1.01)	4.70 (0.97)
Social support (1-7)	4.23 (1.05)	4.37 (1.05)	4.28 (1.10)
Recovery (1-7)	4.41 (1.09)	4.58 (1.01)	4.66 (1.05)
Emotional exhaustion (1-7)	3.73 (1.44)	3.60 (1.44)	3.45 (1.48)

a Percentages are calculated based on the participants who actually responded to each category.

b PTSD: posttraumatic stress disorder.

### Daily Intervention for Active Recovery Intervention

Daily Intervention for Active Recovery is a preventive mobile health intervention designed to improve employee stress management [45]. Daily Intervention for Active Recovery is developed by the research team, with this study being part of an iterative development process. During 4 weeks, users are prompted once daily to engage in a few minutes of self-reflection and psychoeducation. By building a habit of daily reflection and recovery, the intervention aims to support employees in shaping new and effective behavioral responses that mitigate the negative consequences of work stress and thereby prevent stress-related mental health problems.

The design of the Daily Intervention for Active Recovery is rooted in the science of behavior change and makes use of specific strategies to form new habits, such as those outlined in the Behavior Change Technique taxonomy [46]. For instance, self-monitoring of relevant behaviors and emotions serves as a tool for self-insight, which contributes to behavior change. Daily reminders in the app help to maintain engagement and are aligned with habit formation principles. Knowledge-shaping provides users with concrete behavioral or cognitive tools they can use to improve daily recovery from work stress. This combination of self-monitoring, knowledge-shaping, and daily structure is intended to support the formation of new stress management behaviors.

This study investigates the effect of 5 intervention components (Engagement, Demands, Control, Journaling, and Psychoeducation) as well as 3 selected component combinations (Engagement and Demands, Demands and Control, Journaling and Psychoeducation). Table 2 shows each of the 5 components of Daily Intervention for Active Recovery and how they were operationalized, providing an example text used in the intervention.

**Table 2.** Description of Daily Intervention for Active Recovery intervention components including specific examples.

Intervention component	Description	Example text used in Daily Intervention for Active Recovery
Engagement	Prompts participants to reflect on 3 positive emotions they experienced during the last 24 hours related to engagement—asking whether they felt relaxed, engaged, and focused.	During the last 24 h, to what extent have you felt engaged*?* (1 – Not at all, 6 – Very much)
Demands	Prompts participants to reflect on various kinds of job demands they experienced during that day.	To what extent did you have to work under time pressure today? (1 – Not at all, 6 – Very much).
Control	Prompts participants to consider the ways in which they can exert control in their daily lives, both at work and during their free time through job crafting and detachment.	To what extent could you choose your pace of working today? (1 – Not at all, 6 – Very much)
Journaling	Allows participants to journal their thoughts and emotions in free-text form based on prompts related to work-related stress and recovery.	What do you do at work when you feel overwhelmed? (Free-text response)
Psychoeducation	Provides participants with evidence-based information regarding stress and recovery as well as suggesting specific behavioral strategies they may use to recover from daily stress more effectively. Strategies are sampled from the overall literature on stress management, including physical activity, mindfulness, and psychological detachment [24,26,27].	*“*Micro-breaks. Another way to recover from work stress is to take breaks during the day – moments of relaxation when you completely let go of work demands. However, when there is lots going on and we feel stressed, it can be difficult to find the time for longer breaks. Perhaps there are no clear opportunities for resting in between tasks. In these cases, it is especially important to do short interruptions – micro-breaks – to sit down, close your eyes, and breathe deeply for a minute*.”*

Participants were randomized to experimental conditions using blocked randomization with a block size of 32 participants, ensuring equal distribution of participants across all conditions. The randomization sequence was computer-generated. All aspects of the randomization process, participant enrollment, sequence generation, and condition assignment were conducted by the first author. Participants were blinded to their experimental condition, not knowing what intervention version they had received.

### Study Design

**Figure 1.**
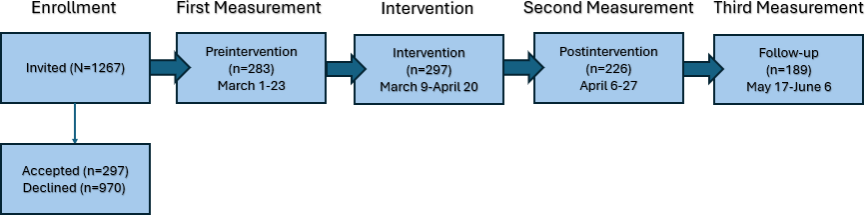
Flowchart showing the timeline of measures and intervention. Note: Some dates overlap or suggest a longer time interval due to participants starting the study at slightly different time points. There were 297 participants who took part in the study, where 283 completed the full preintervention measure.

The study followed the preregistered design as described in ClinicalTrials.gov (registration NCT04719351) [47]. Data were collected between March 2021 and June 2021, with 3 identical surveys sent out during this time period (as depicted in Figure 1) : (1) preintervention measured immediately before the intervention, (2) postintervention measured immediately after the intervention, and (3) follow-up measured 1 month after finishing the intervention. All data were collected through the mobile app Daily Intervention for Active Recovery.

**Figure 2.**
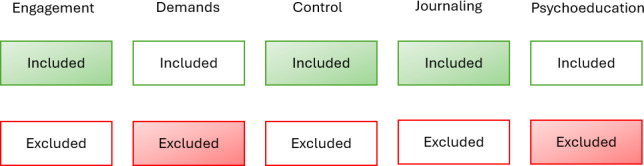
Illustration of a sample experimental condition, a specific intervention version offered to a set of participants. Note: Each component—Engagement, Demands, Control, Journaling, Psychoeducation—is either included or excluded from a particular experimental condition. In this particular experimental condition, the intervention version includes components Engagement, Control, and Journaling while excluding components Demands and Psychoeducation from the intervention version. All possible permutations of these component inclusion and exclusion are used, resulting in 32 experimental conditions.

The study used a full factorial experimental design examining the effect of 5 factors. Each factor (the intervention components as described in Table 2) has two levels: (1) included or (0) excluded from the intervention. Inclusion and exclusion of each component, combined in all possible permutations, resulted in 2^5^=32 experimental conditions or versions of the intervention. Each component was present in the intervention for half the participants, so the effect of that component can be measured by comparing outcomes between groups of participants who received the component versus those who did not receive it (as depicted in Figure 2).

### Measures

For complete details on all measures, see supplementary materials. Primary outcomes include measures of burnout, emotional exhaustion, and stress. Burnout was measured using the Oldenburg Burnout Inventory (OLBI) [48,49]. Emotional exhaustion was measured using the Shirom-Melamed Burnout Questionnaire (SMBQ) [50]. Stress was measured using the Perceived Stress Scale (PSS) [51]. Secondary outcomes include measures of depression, anxiety, posttraumatic stress, recovery, job crafting, mindfulness, and social support. Depression was measured using the Patient Health Questionnaire (PHQ) [52]. Anxiety was measured using the Generalized Anxiety Disorder (GAD) questionnaire [53]. Posttraumatic stress disorder symptoms were measured using selected items from the Posttraumatic Stress Disorder Checklist (PCL) [54]. Mindfulness was measured using the Mindful Attention Awareness Scale (MAAS) [55]. Recovery was measured using 2 subscales from the Recovery Experience Questionnaire (REQ) [56]. Job crafting was measured using the REQ and Job Crafting Questionnaire (JCQ) [56,57]. Social support was measured using the JCQ [57].

Analyses investigating differences in mental health between demographic groups indicate that assistant nurses, those who have worked 1‐5 years, men, and younger participants report higher levels of stress-related health problems at the preintervention time point. Assistant nurses had higher levels of emotional exhaustion, burnout, depression, and posttraumatic stress disorder relative to most other occupational groups. Men reported higher levels of anxiety, emotional exhaustion, and depression than women. Those who had worked 1‐5 years had higher levels of anxiety, stress, emotional exhaustion, and burnout than those who had worked more than 10 years. Lower age was correlated with poorer mental health on nearly all measures. Please see Tables S1.1-S2.2 in Multimedia Appendix 1 for full output of these analyses.

### Statistical Analysis

All data were analyzed with R (R Core Team) using the lme4, datawizard, and Tidyverse packages [58-61]. Anonymized versions of the dataset and all code used for statistical analysis are available via Open Science Framework [62].

This study uses multilevel piecewise growth models to estimate effects of the intervention [63]. Piecewise growth models are especially suited for capturing complex growth trajectories in longitudinal data. These models divide the time variable into distinct phases to account for shifts that may occur in growth trajectories, allowing different change rates at specific points in time.

Time was modeled on the within-level, each participant having repeated measures of the same variable at 3 time points. In a piecewise growth model, multiple time variables are included as predictors to model how outcomes change over different time intervals. A first-time variable refers to change that happens during the intervention, coding the preintervention time point as −1 and post- and follow-up time points as 0. A second time variable refers to change that occurs after intervention, coding pre- and postintervention time points as 0, while the follow-up time point is coded as 1.

Components, Engagement, Demands, Control, Journaling, and Psychoeducation were coded 1 or 0, indicating whether this component was included or excluded in the experimental condition.

Each multilevel model included 1 or 2 components (estimating the effect of single components or the interaction of 2 components), both time variables (change during intervention and change after intervention), with the individual as the clustering variable. All interactions between components and time were included in the model. Separate models were constructed for each outcome variable. Initially, single-component models were estimated before calculating the interaction effects of each two-component combination.

All outcome variables were standardized using the standardize function from the “datawizard” package. To interpret the analyses, it was of primary interest to look at interaction effects between components and time. A statistically significant interaction between component and time indicates that participants who received this component experienced a difference (ie, improvement or worsening, depending on the sign of the effect) in an outcome over a given time frame (ie, during or after the intervention) compared with participants who did not receive this component.

In addition, since not all participants responded at all measures, we conducted dropout and missingness analysis to investigate any differences between participants based on their response patterns. We conducted comparisons between participants who completed all measures and those who did not respond at all time points. The Wilcoxon test was used for continuous variables (age and mental health measures), while the chi-square test was used on categorical variables (gender, tenure, and occupation) at the preintervention time point. Furthermore, a Little test was conducted to analyze whether data were missing completely at random or whether there was some systematic pattern in the missing data.

All data were analyzed using the intention-to-treat principle, in which all participants and data points are included in the analyses regardless of the extent to which participants adhered to the intended intervention protocol. The multilevel models employed in this analysis are also well-suited to account for missing data.

### Ethical Considerations

Ethical approval was granted by the Swedish Ethical Review Authority (reference numbers 2020‐01795 and 2022-01546-02). Participants received an informed consent form with the complete study procedure and clearly stated information that participation is voluntary and that they may drop out of the study at any time. No compensation or incentives were offered for participation. All data have been pseudonymized so that only the research team can access personal information. All data made publicly available through open science principles have been completely anonymized.

## Results

### Dropout Analysis

Dropout analysis indicates one significant difference that participants who completed all measures were 2.80 years older (*P*=.03) than participants who did not respond at all time points. Little Test (χ^2^=786.27; *P*=.57) was statistically insignificant and thus fails to reject the hypothesis that data are missing completely at random. See Tables S3.1-S3.3 in Multimedia Appendix 1 for complete output of these analyses.

### Component Effects Analysis

To determine the most effective set of components for improving health outcomes, we compared the magnitude of the effect that each component and each 2-way interaction of components had on health outcomes. The strongest component effects are presented below, as determined by a 0.4 threshold on estimated standardized effect. First, component effects during the intervention are presented, followed by component effects after the intervention. Complete output from the statistical analyses is available in Appendices “Effects of 1 component predictor” and “Effects of 2 component predictors” with graphical representation in Appendices “Effects of 1 component predictor, Graphs” and “Effects of 2 component predictors, Graphs” that can be found in the Open Science Framework repository [62].

**Figure 3.**
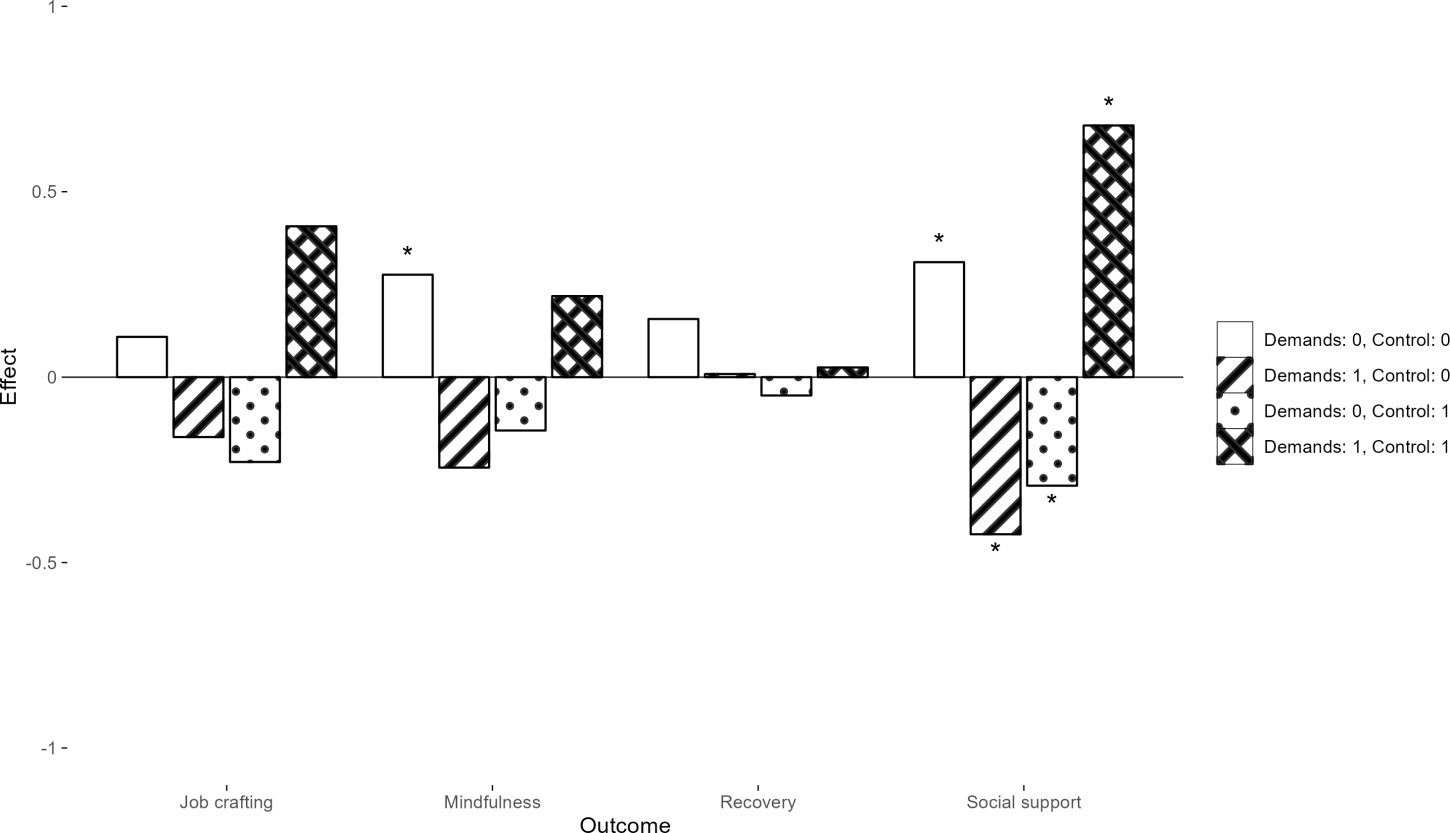
Estimated intervention effects on stress-related health outcomes from a factorial experiment among Swedish health care workers showing change during the intervention (change that happens between pre- and post/follow-up measure). Note: Each bar shows the estimated standardized effects during the intervention of participants who received a specific combination of Demands and Control components. ^∗^*P*<.05.

No single component had a standardized effect on any of the outcomes above the 0.4 threshold. When considering the effects of 2-component interactions during the intervention, including the Demands and Control components led to an increase in social support (β=0.68; *P*<.001) while an increase in job crafting (β=0.41; *P*=.06) was not statistically significant. These results are presented in Figure 3. Including Journaling and Psychoeducation components as part of the intervention led to an increase in social support (β=0.41; *P*=.04).

**Figure 4.**
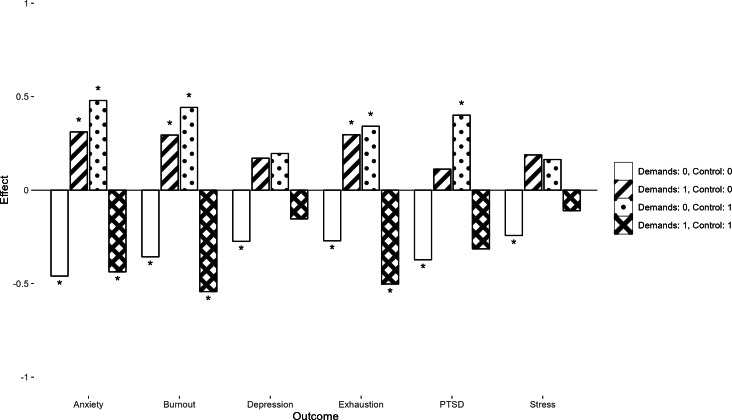
Estimated intervention effects on stress-related health outcomes from a factorial experiment among Swedish health care workers showing change after the intervention (change that happens between pre/post measure and follow-up measure). Note: Each bar shows the standardized estimated effect after the intervention of participants who received a specific combination of Demands and Control components. PTSD: posttraumatic stress disorder. ^∗^*P*<.05.

When considering the effects of 2-component interactions after the intervention, including the Demands and Control components led to a decrease in symptoms of emotional exhaustion (β=−0.50; *P*=.005), burnout (β=−0.54; *P*=.004), and anxiety (β=−0.44; *P*=.04), while including neither the Demands nor the Control component in the intervention led to a decrease in anxiety symptoms (β=−0.46; *P*<.001). Notably, including one of these components without the other often led to a negative effect on health outcomes. These results are presented in Figure 4.

Mindfulness after the intervention increased when both the Engagement and Demands components (β=0.72; *P*=.001) were included as well as when the Journaling and Psychoeducation components were included (β=0.46; *P*=.04).

To control for demographic variables as potential confounders, we included age, gender, tenure, and occupation as covariates in the above-mentioned analyses. These analyses indicate that demographic variables did not influence the reported results. The complete output is shown in Tables S4.1-S4.3 in Multimedia Appendix 1

## Discussion

### Principal Findings

Our findings indicate that combining Demands and Control components yields the strongest benefits for mental health outcomes. Similarly, the synergy between Demands and Engagement as well as between Journaling and Psychoeducation demonstrates improvements in participant health relative to other types of content. These patterns highlight the importance of integrating self-reflective practices with action-oriented strategies in DBCIs targeting workplace stress. Notably, health improvements were more pronounced following the intervention than during, aligned with our intention that DBCIs may foster sustainable behavior and health changes that continue beyond the end of the intervention.

### Intervention Component Effects

The overall aim of the study was to identify which components or combinations of components are most effective at preventing symptoms of stress-related health problems. The strength of effects was overall larger for component interactions than for single components, suggesting it is worthwhile to investigate how to combine different components to best support intended health outcomes. For all explored interactions, Engagement and Demands, Demands and Control, Journaling and Psychoeducation, including both of the components in the intervention generally led to better health outcomes compared to including only 1 component by itself.

The most evident effect was noted for the interaction between the Demands and Control components (see Figures3  4). Stress-related outcomes, such as exhaustion, burnout, and anxiety, seem to improve from including both components in the intervention, while including one of these components without the other often had a negative effect. It is possible that aspects of the work environment should optimally be seen as a balance between demands and control, where one without the other may be detrimental.

For instance, focusing exclusively on control aspects without sufficient reflection on job demands may not be helpful in supporting workers in shaping a better work environment. Control strategies, such as job crafting and detachment, may be counterproductive when participants have not first identified and reflected on their specific work demands. By not developing an awareness of the workplace stressors to manage in the first place, implementing these strategies may lead to misaligned or ineffective coping attempts. Given that control strategies also require expediting effort, if these strategies are misaligned with work demands, they may have the opposite effect of draining already limited resources.

A complementary interpretation is that solely reflecting on job demands may lead to an excessive focus on negative aspects of work. This may be related to rumination (“pervasive, recurrent, negative thoughts”) or negative work reflection (“thinking about the bad sides of one’s job”), which is known to have a detrimental effect on mental health [64]. Supplementing job demands reflection with the Control component, which includes questions about detachment and job crafting, is likely to remind participants of their ability to shape their work environment and thus reframe the reflection in a more constructive light. Psychological detachment, mentally disengaging from work during free time, may function as an antidote to work-related rumination by helping participants to focus on their leisure activities [36]. Likewise, job crafting provides an active orientation to job demands that is more fruitful than being stuck in recurrent, negative thoughts. In this way, the Control component may decrease the risk of rumination and instead lead to a more action-oriented attitude.

A similar relationship can be seen in the interaction between the Demands and Engagement components. The Engagement component helped participants focus on positive emotions they experienced as part of the working day, and this may contribute to reappraising their reflection of job demands. Rather than ruminating on work problems or engaging in negative work reflection, paying attention to engagement and related feelings may contribute to a more positive outlook on work [65]. In effect, this combination is similar to positive work reflection, and our results corroborate findings on the effect of positive work reflection interventions, which have been found to improve mental health and job satisfaction [33,66].

An underlying pattern may be that optimal intervention effects are observed when components that raise awareness are combined with components that frame insights in a fruitful way or suggest actionable strategies. For instance, reflecting on job demands through a positive lens or while considering job crafting possibilities seems to be superior to simply reflecting on challenges in the working environment. Combining Journaling (self-reflection and insight) with Psychoeducation (knowledge and advice on actionable strategies) is aligned with this synergy and indeed shows similar interaction effects.

The value of integrating these aspects in an intervention is supported by therapeutic modalities such as CBT, where journaling is often accompanied by techniques, such as cognitive reappraisal or behavioral activation [67,68]. Our findings also map onto previous evidence suggesting that combining several behavior change techniques leads to improved intervention effects [68,69]. More specifically, Michie et al [46] found that intervention effects are significantly improved when combining self-monitoring with other behavior change techniques, also in line with our data.

### Time Effects

Another noteworthy observation regards how intervention effects differ between the different time frames. Stronger positive effects are generally observed after relative to during the intervention, which is aligned with how the intervention is thought to function. Rather than providing a temporary boost, the intention is to support participants in creating long-term changes in how they respond to and manage stress that are sustained beyond the duration of the intervention. This goal of generating long-lasting effects from digital and internet-based interventions is indeed possible, as studies report positive health outcomes at 6-month, 1-year, and even longer-term follow-ups [70-72].

One explanation for observed effects being stronger after the intervention is that these behavioral and health changes simply take some time to come into effect. The mechanisms of the intervention, self-reflection, learning new behavioral strategies, etc, are gradual processes that naturally evolve over weeks and months. It takes a while to integrate insights gathered from self-reflection, and it is well-known that changing behaviors and developing new habits require both practice and time [73,74]. To further support sustained behavioral and health changes over time, there are several features that may be included in the intervention. Continued self-monitoring, follow-up “booster” sessions, and various kinds of accountability or peer support systems may be important for maintaining engagement over longer time periods [75,76].

### Key Takeaways

Optimal intervention outcomes are observed when combining self-reflection with beneficial framing or concrete action strategies. Simply prompting participants to engage in self-reflection may not be sufficient; it is also necessary to help participants integrate these insights into empowering beliefs and behaviors. By integrating aspects, such as self-reflection, helpful framing, and actionable steps, the intervention uses several avenues for supporting participants in improving their ways of managing stress.

Given the key role of self-monitoring in supporting behavior change, components, including self-monitoring (Demands or Engagement), should certainly make up part of the intervention. The positive interaction effect between the Demands and Control components was especially pronounced and should be kept as part of the intervention. Finally, Psychoeducation, as the main component supporting knowledge-shaping and giving concrete behavioral suggestions, may also be valuable as part of a digital behavior change intervention for work stress.

### Limitations

A primary limitation is related to the interpretation of estimated intervention effects. Most effects indicate relatively small impacts on health outcomes; however, since this is a preventive intervention rather than a treatment, small effects do not necessarily imply the intervention is ineffective [76]. At the same time, modest effects also make it difficult to assess whether the intervention functions as intended. To gain better insights, it can be beneficial to include more proximal and process-related outcomes aligned with the specific aims of the intervention. For example, how many times a person used a recovery strategy could serve as an early indicator of the intervention’s effectiveness.

A notable methodological limitation of this study is the use of an arbitrary threshold of 0.4 as a cutoff for interpreting estimated effects. This threshold was selected to narrow down the extensive set of analyses and focus on the most substantial results. Given the exploratory nature of this study, it was considered appropriate to use an arbitrary threshold to highlight the strongest effects. However, future research on the efficacy of the intervention should use more rigorously justified criteria for evaluating component effects, potentially by considering effects that have a meaningful impact on health or behavioral outcomes [77,78].

Another limitation of this study was the progressive participant dropout across measurement time points (see Figure 1), which may decrease statistical power and potentially bias our results. For context, participant engagement in digital intervention research is notoriously problematic, and our attrition rates align with patterns from similar studies in this field [79,80]. We have handled this issue by conducting dropout analyses and using appropriate statistical models that are well-suited to handle missing data. Given that dropout analyses revealed no systematic differences between participants based on response patterns (apart from slight differences in age), the findings of our study likely remain valid despite incomplete follow-ups.

An important limitation concerns our follow-up period, which extended only 1 month after intervention completion. While we observed improved effects at this time point, longer-term follow-up would be necessary to determine whether intervention benefits persist over extended periods or gradually diminish. Future research should incorporate follow-up assessments at further time points to better understand the durability of intervention effects and identify whether repeated exposure to the intervention should be offered to sustain improvements in stress-related outcomes.

Another limitation is that data were collected during the COVID-19 pandemic, a time of significant workload and stress for health care workers. While this could be positive given that the intervention is intended to help employees manage stress, the unique working conditions of this time may limit the replicability and ecological validity of the research.

### Future Directions

This study is part of an ongoing user-centered iterative development process of Daily Intervention for Active Recovery[45]. In line with the optimization phase of the MOST, this study was intended as an exploratory investigation to guide future decision-making regarding what components to include in the intervention. Rather than evaluating the effectiveness of the intervention as a whole, this is a formative assessment aimed at refining the intervention by determining what components to incorporate.

The next part of MOST, the evaluation phase, involves testing the efficacy of an optimized version of the intervention [43]. The findings of this study could inform how to further refine Daily Intervention for Active Recovery so that an improved version can be tested in a randomized controlled trial. Importantly, future efficacy studies should involve long-term follow-up measures that are beyond 1 month after the end of the intervention. Given that the aim of Daily Intervention for Active Recovery is to have long-term preventive effects, it would be important that a randomized controlled trial include outcome measures at 1 year or longer past the end of the intervention.

Future research could also investigate how to more effectively weave together various components of the intervention. As an example, the positive interaction effects between the Demands and Control components could potentially be further improved by having the specific reflection for each component relate to the same topic so that the Control reflection is directly related to the Demands reflection of that day. For instance, if Demands reflection regards challenges with handling workload, the Control component may suggest a reflection or strategy for how one can deal with excessive workload.

### Conclusion

This study provides preliminary evidence on what components should be included in digital behavior change interventions for work stress. Self-monitoring mood and its relationship to work factors is an integral part of preventive interventions, serving to initiate a self-reflection process. Optimally, self-reflection should be balanced with aspects of the intervention that provide evidence-based knowledge to users such that they may integrate insights into actionable behavior changes. Future research could further investigate this relationship to clarify how to best integrate these components to further improve digital behavior change interventions.

## Supplementary material

10.2196/71032 Multimedia Appendix 1 Supplementary materials that show additional information, including outcome measure details, dropout analyses, subgroup analyses, and models with control variables.

## Abbreviations

CBT: cognitive behavioral therapy
DBCI: Digital Behavior Change Interventions
GAD: Generalized Anxiety Disorder
JCQ: Job Crafting Questionnaire
MAAS: Mindful Attention Awareness Scale
MOST: Multiphase Optimization Strategy
OLBI: Oldenburg Burnout Inventory
PCL: Posttraumatic Stress Disorder Checklist
PHQ: Patient Health Questionnaire
PSS: Perceived Stress Scale
REQ: Recovery Experience Questionnaire
SMBQ: Shirom-Melamed Burnout Questionnaire
