# Supplementary material for: Optimizing Intervention Components of a Preventive Stress Management mHealth Intervention for Health Care Workers: Experimental Factorial Study
Source: JMIR Form Res. 2025 Aug 25;9:e71032. doi: 10.2196/71032 (PMC12377794; doi:10.2196/71032)

Outcome measures

Primary outcomes:

Burnout was measured using the Oldenburg Burnout Inventory (OLBI), an instrument designed to measure exhaustion and disengagement from work (Halbesleben & Demerouti, 2005). This study used a Swedish translation with a subset of 7 items (Gustavsson et al., 2010; Peterson et al., 2011). The instrument indicated high internal consistency at pre-, post-, and follow-up measures (α ≥ 0.88). Items (e.g., "after work I often feel tired and exhausted") are scored on a 4-point ordered categories scale (1 = Strongly disagree, 4 = Strongly agree).

Emotional exhaustion was measured using the Shirom-Melamed Burnout Questionnaire (SMBQ), an instrument designed to measure burnout (Shirom & Melamed, 2006). This study uses a Swedish translation of SMBQ-6, a subset of six items (Sundström et al., 2022). The instrument indicated high internal consistency at pre-, post-, and follow-up measures (α ≥ 0.92). Items (e.g., "I feel my batteries are empty") are scored on a 7-point ordered categories scale (1 = Very rarely, 7 = Very frequently).

Stress was measured using the Perceived Stress Scale (PSS), an instrument designed to measure how stressful one appraised life events (Cohen et al., 1983). This study uses Swedish translation with 10 items. (Nordin & Nordin, 2013). The instrument indicated high internal consistency at pre-, post-, and follow-up measures (α ≥ 0.88). Items (e.g., "how often have you been able to control irritations in your life") are scored on a 5-point ordered categories scale (1 = Never, 5 = Very often).

Secondary outcomes:

Depression was measured using the Patient Health Questionnaire (PHQ-2) a two-item screening tool designed to detect depression and anhedonia (Kroenke et al., 2003). This study uses a Swedish translation (Nordin et al., 2013). The instrument indicated acceptable internal consistency at pre-, post-, and follow-up measures with pearson correlation (r = 0.64, r = 0.72, r = 0.69). Items (e.g., "feeling down, depressed, or hopeless") were scored on a4-point ordered categories scale (1 = Not at all, 4 = Nearly every day).

Anxiety was measured using the Generalized Anxiety Disorder (GAD) questionnaire, a 7-item instrument designed to assess generalized anxiety disorder (Spitzer et al., 2006). This study uses a Swedish translation (Johansson et al., 2013). The instrument indicated high internal consistency at pre-, post-, and follow-up measures (α ≥ 0.86). Items (e.g., "Feeling nervous, anxious, or on edge") were scored on a 4-point ordered categories scale (1 = Not at all, 4 = Nearly every day) (Johansson et al., 2013).

Post-traumatic stress disorder (PTSD) symptoms were measured using the Posttraumatic Stress Disorder Checklist (PCL-5), one of the most widely used self-reported measures of PTSD (Blevins et al., 2015). This study uses a Swedish translation with a subset of three items (Sveen et al., 2016). The instrument indicated satisfactory internal consistency at pre-, post-, and follow-up measures (α ≥ 0.75) Items (e.g., "how much were you been bothered by: "Repeated, disturbing, and unwanted memories of the stressful experience?") are rated on a 5-point ordered categories scale (0 = Not at all, 4 = Extremely).

Mindfulness was measured using Mindful Attention Awareness Scale (MAAS), a widely used measure of mindfulness (Brown & Ryan 2006). This study uses a subset of six items translated into Swedish focusing on emotional awareness (Hansen et al., 2009). The instrument indicated high internal consistency at pre-, post-, and follow-up measures (α ≥ 0.87). Items (e.g., "I could be experiencing some emotion and not be conscious of it until later") are rated on a 6-point ordered categories scale (1 = Very frequently, 6 = Very rarely).

Recovery was measured using two subscales from the Recovery Experience Questionnaire (REQ) - psychological detachment and (Sonnentag et al., 2007). The study used a Swedish translation (Almén et al., 2018). The instrument indicated satisfactory internal consistency at pre-, post-, and follow-up measures (α ≥ 0.77). Each subscale includes four items (e.g., "On my free time, I don´t think about work at all") scored on a 7-point ordered categories scale (1 = Almost never, 7 = Almost always).

Job crafting was measured using the Control subscale from the Recovery Experience Questionnaire (REQ) and the Task Crafting subscale from the Job Crafting Questionnaire (JCQ) (Sonnentag et al., 2007; Slemp & Vella-Brodrick, 2013). The instrument indicated rather low internal consistency at pre- measure (α = 0.67) while acceptable at post- (α = 0.73) and follow-up measures. (α = 0.75). Task Crafting subscale includes five items (e.g., "Give preference to work tasks that suit your skills or interests") scored on a 7-point ordered categories scale (1 = Almost never, 5 = Almost always). The Control subscale includes 4 items (e.g. "On my free time, I determine for myself how I will spend my time") scored on a 5-point ordered categories scale (1 = Not at all, 5 = Very much).

Social support was measured using the Relation Crafting subscale from the Job Crafting Questionnaire (JCQ), an instrument designed to measure the degree to which people engage with their social context for emotional support (Slemp & Vella-Brodrick, 2013). The instrument indicated satisfactory internal consistency at pre-, post-, and follow-up measures (α ≥ 0.71). The study uses a Swedish translation of five items (e.g., "Make an effort to get to know people well at work") scored on a 7-point ordered categories scale (1 = Almost never, 7 = Almost always).

References:

Almén, N., Lisspers, J., Öst, L.-G., & Sundin, Ö. (2020). Behavioral stress recovery management intervention for people with high levels of perceived stress: A randomized controlled trial. International Journal of Stress Management, 27(2), 183–194. https://doi.org/10.1037/str0000140

Almén, N., Lundberg, H., Sundin, Ö., & Jansson, B. (2018). The reliability and factorial validity of the Swedish version of the Recovery Experience Questionnaire. Nordic Psychology, 70(4), 324–333. https://doi.org/10.1080/19012276.2018.1443280

Blevins, C. A., Weathers, F. W., Davis, M. T., Witte, T. K., & Domino, J. L. (2015). The Posttraumatic Stress Disorder Checklist for DSM-5 (PCL-5): Development and Initial Psychometric Evaluation: Posttraumatic Stress Disorder Checklist for DSM-5. Journal of Traumatic Stress, 28(6), 489–498. https://doi.org/10.1002/jts.22059

Cohen, S., Kamarck, T., & Mermelstein, R. (1983). A Global Measure of Perceived Stress. Journal of Health and Social Behavior, 24(4), 385. https://doi.org/10.2307/2136404

Gustavsson, J. P., Hallsten, L., & Rudman, A. (2010). Early career burnout among nurses: Modelling a hypothesized process using an item response approach. International Journal of Nursing Studies, 47(7), 864–875. https://doi.org/10.1016/j.ijnurstu.2009.12.007

Halbesleben, J. R. B., & Demerouti, E. (2005). The construct validity of an alternative measure of burnout: Investigating the English translation of the Oldenburg Burnout Inventory. Work & Stress, 19(3), 208–220. https://doi.org/10.1080/02678370500340728

Hansen, E., Lundh, L., Homman, A., & Wångby-Lundh, M. (2009). Measuring Mindfulness: Pilot Studies with the Swedish Versions of the Mindful Attention Awareness Scale and the Kentucky Inventory of Mindfulness Skills. Cognitive Behaviour Therapy, 38(1), 2–15. https://doi.org/10.1080/16506070802383230

Johansson, R., Carlbring, P., Heedman, Å., Paxling, B., & Andersson, G. (2013). Depression, anxiety and their comorbidity in the Swedish general population: Point prevalence and the effect on health-related quality of life. PeerJ, 1, e98. https://doi.org/10.7717/peerj.98

Kroenke, K., Spitzer, R. L., & Williams, J. B. W. (2003). The Patient Health Questionnaire-2: Validity of a Two-Item Depression Screener. Medical Care, 41(11), 1284–1292. https://doi.org/10.1097/01.MLR.0000093487.78664.3C

Nordin, M., & Nordin, S. (2013). Psychometric evaluation and normative data of the Swedish version of the 10-item perceived stress scale. Scandinavian Journal of Psychology, 54(6), 502–507. https://doi.org/10.1111/sjop.12071

Nordin, S., Palmquist, E., & Nordin, M. (2013). Psychometric evaluation and normative data for a Swedish version of the Patient Health Questionnaire 15-Item Somatic Symptom Severity Scale: Health and Disability. Scandinavian Journal of Psychology, 54(2), 112–117. https://doi.org/10.1111/sjop.12029

Peterson, U., Bergström, G., Demerouti, E., Gustavsson, P., Åsberg, M., & Nygren, Å. (2011). Burnout Levels and Self-Rated Health Prospectively Predict Future Long-Term Sickness Absence: A Study Among Female Health Professionals. Journal of Occupational & Environmental Medicine, 53(7), 788–793. https://doi.org/10.1097/JOM.0b013e318222b1dc

Shirom, A., & Melamed, S. (2006). A comparison of the construct validity of two burnout measures in two groups of professionals. International Journal of Stress Management, 13(2), 176–200. https://doi.org/10.1037/1072-5245.13.2.176

Slemp, G. R., & Vella-Brodrick, D. A. (n.d.). The job crafting questionnaire: A new scale to measure the extent to which employees engage in job crafting.

Sonnentag, S., & Fritz, C. (2007). The Recovery Experience Questionnaire: Development and validation of a measure for assessing recuperation and unwinding from work. Journal of Occupational Health Psychology, 12(3), 204–221. https://doi.org/10.1037/1076-8998.12.3.204

Spitzer, R. L., Kroenke, K., Williams, J. B. W., & Löwe, B. (2006). A Brief Measure for Assessing Generalized Anxiety Disorder: The GAD-7. Archives of Internal Medicine, 166(10), 1092. https://doi.org/10.1001/archinte.166.10.1092

Sundström, A., Söderholm, A., Nordin, M., & Nordin, S. (2022). Construct validation and normative data for different versions of the Shirom-Melamed burnout questionnaire/measure in a Swedish population sample. Stress and Health, smi.3200. https://doi.org/10.1002/smi.3200

Sveen, J., Bondjers, K., & Willebrand, M. (2016). Psychometric properties of the PTSD Checklist for DSM-5: A pilot study. European Journal of Psychotraumatology, 7(1), 30165. https://doi.org/10.3402/ejpt.v7.30165

1. Baseline Subgroup Analyses

Presents baseline (pre-intervention) differences in outcome measures across demographic subgroups.

Statistical tests used:

• Kruskal-Wallis test for occupation and tenure (non-parametric alternative to one-way ANOVA)

• Wilcoxon rank-sum test for gender (non-parametric alternative to t-test)

• Spearman correlation for age (non-parametric correlation)

Significance levels: * $p < 0.05$, ** $p < 0.01$, *** $p < 0.001$

1.1 Occupation Differences

Outcome	χ^2	p_value	Assistant nurse	Nurse	Physician	Admin	Other
Anxiety	9.04	0.06	2.12	1.74	1.57	1.74	1.55
Stress	7.53	0.11	2.91	2.48	2.39	2.53	2.37
Mindfulness	4.04	0.40	3.70	2.95	2.91	2.92	2.91
Emotional Exhaustion	10.92	0.03*	4.61	3.85	3.45	3.97	3.52
Burnout	14.93	0.005**	2.90	2.54	2.28	2.48	2.28
Depression	21.11	0.000***	2.54	1.75	1.53	1.84	1.63
Recovery	2.66	0.62	4.56	4.38	4.28	4.24	4.54
Job Crafting	14.40	0.006**	4.85	4.93	4.69	5.20	5.10
Ptsd	11.31	0.02*	2.74	2.18	1.96	2.11	1.89
Social Support	0.70	0.95	4.01	4.27	4.30	4.19	4.23

1.2 Gender Differences

Outcome	W	p_value	Female	Male	Mean Difference
Anxiety	3,801	0.03*	1.51	1.71	0.20
Stress	4,123	0.18	2.33	2.49	0.15
Mindfulness	3,920	0.07	2.64	3.03	0.39
Emotional Exhaustion	3,666	0.009**	3.24	3.84	0.60
Burnout	4,246	0.10	2.28	2.45	0.18
Depression	3,502	0.004**	1.48	1.78	0.30
Recovery	5,202	0.38	4.54	4.37	-0.17
Job Crafting	4,081	0.05*	4.76	5.03	0.27
Ptsd	4,363	0.51	1.99	2.09	0.10
Social Support	4,112	0.02*	3.92	4.31	0.39

1.3 Tenure Differences

Outcome	χ^2	p_value	< 1 Year	1-5 Years	5-10 Years	10+ Years
Anxiety	15.09	0.002**	1.52	1.92	1.69	1.59
Stress	9.53	0.02*	2.15	2.69	2.44	2.40
Mindfulness	11.87	0.008**	3.02	3.46	3.00	2.79
Emotional Exhaustion	12.13	0.007**	3.83	4.30	3.80	3.51
Burnout	15.18	0.002**	2.30	2.73	2.43	2.32
Depression	3.89	0.27	1.62	1.93	1.75	1.67
Recovery	4.06	0.25	4.65	4.15	4.39	4.46
Job Crafting	6.96	0.07	4.61	4.92	4.82	5.08
Ptsd	2.69	0.44	1.96	2.24	1.92	2.07
Social Support	1.30	0.73	4.15	4.08	4.15	4.28

1.4 Age Correlations

Age statistics: Mean = 45.66 (SD = 12.01), Range = 24-85

Spearman correlation coefficients (rho) between age and outcome variables:

Outcome	n	rho	p_value
Anxiety	265	-0.33	0.000***
Stress	263	-0.26	0.000***
Mindfulness	264	-0.27	0.000***
Emotional Exhaustion	266	-0.31	0.000***
Burnout	268	-0.35	0.000***
Depression	265	-0.16	0.01*
Recovery	266	0.19	0.002**
Job Crafting	268	0.20	0.001**
Ptsd	264	-0.13	0.03*
Social Support	270	0.05	0.38

2. Post-hoc Analyses

Post-hoc pairwise comparisons using Dunn's test with Bonferroni correction for significant Kruskal-Wallis results.

2.1Occupation Post-hoc Tests

2.1.1 Emotional Exhaustion - Pairwise Comparisons

Comparison	Z	p-value (adj)	Mean Diff
Admin - Assistant nurse	-1.48	0.69	-0.64
Admin - Nurse	0.68	1.00	0.12
Assistant nurse - Nurse	1.98	0.24	0.76
Admin - Other	1.85	0.32	0.45
Assistant nurse - Other	2.73	0.03*	1.09
Nurse - Other	1.29	0.99	0.33
Admin - Physician	1.74	0.41	0.52
Assistant nurse - Physician	2.66	0.04*	1.16
Nurse - Physician	1.23	1.00	0.40
Other - Physician	0.17	1.00	0.06

2.1.2 Burnout - Pairwise Comparisons

Comparison	Z	p-value (adj)	Mean Diff
Admin - Assistant nurse	-1.78	0.38	-0.42
Admin - Nurse	-0.33	1.00	-0.06
Assistant nurse - Nurse	1.63	0.51	0.35
Admin - Other	1.80	0.36	0.20
Assistant nurse - Other	3.05	0.01*	0.62
Nurse - Other	2.43	0.07	0.26
Admin - Physician	1.55	0.60	0.20
Assistant nurse - Physician	2.85	0.02*	0.62
Nurse - Physician	2.03	0.21	0.26
Other - Physician	0.01	1.00	0.00

2.1.3 Depression - Pairwise Comparisons

Comparison	Z	p-value (adj)	Mean Diff
Admin - Assistant nurse	-2.66	0.04*	-0.70
Admin - Nurse	1.26	1.00	0.09
Assistant nurse - Nurse	3.59	0.002**	0.79
Admin - Other	1.83	0.33	0.21
Assistant nurse - Other	3.98	0.000***	0.91
Nurse - Other	0.58	1.00	0.12
Admin - Physician	2.25	0.12	0.30
Assistant nurse - Physician	4.19	0.000***	1.00
Nurse - Physician	1.22	1.00	0.22
Other - Physician	0.77	1.00	0.09

2.1.4 Job Crafting - Pairwise Comparisons

Comparison	Z	p-value (adj)	Mean Diff
Admin - Assistant nurse	1.55	0.61	0.35
Admin - Nurse	1.76	0.39	0.27
Assistant nurse - Nurse	-0.45	1.00	-0.08
Admin - Other	0.72	1.00	0.10
Assistant nurse - Other	-1.18	1.00	-0.25
Nurse - Other	-1.28	1.00	-0.17
Admin - Physician	3.36	0.004**	0.51
Assistant nurse - Physician	0.81	1.00	0.16
Nurse - Physician	1.94	0.26	0.24
Other - Physician	3.14	0.008**	0.41

2.1.5 Ptsd - Pairwise Comparisons

Comparison	Z	p-value (adj)	Mean Diff
Admin - Assistant nurse	-2.31	0.10	-0.63
Admin - Nurse	-0.08	1.00	-0.07
Assistant nurse - Nurse	2.36	0.09	0.56
Admin - Other	1.19	1.00	0.22
Assistant nurse - Other	3.22	0.006**	0.85
Nurse - Other	1.46	0.71	0.29
Admin - Physician	0.65	1.00	0.15
Assistant nurse - Physician	2.75	0.03*	0.78
Nurse - Physician	0.79	1.00	0.21
Other - Physician	-0.45	1.00	-0.07

2.2. Tenure Post-hoc Tests

2.2.1 Anxiety - Pairwise Comparisons

Comparison	Z	p-value (adj)	Mean Diff
< 1 Year - 1-5 Years	-1.81	0.21	-0.41
< 1 Year - 10+ Years	-0.24	1.00	-0.07
1-5 Years - 10+ Years	3.82	0.000***	0.34
< 1 Year - 5-10 Years	-0.65	1.00	-0.18
1-5 Years - 5-10 Years	2.17	0.09	0.23
10+ Years - 5-10 Years	-0.93	1.00	-0.11

2.2.2 Stress - Pairwise Comparisons

Comparison	Z	p-value (adj)	Mean Diff
< 1 Year - 1-5 Years	-2.12	0.10	-0.54
< 1 Year - 10+ Years	-1.01	0.93	-0.25
1-5 Years - 10+ Years	2.76	0.02*	0.29
< 1 Year - 5-10 Years	-1.12	0.79	-0.29
1-5 Years - 5-10 Years	1.84	0.20	0.25
10+ Years - 5-10 Years	-0.36	1.00	-0.04

2.2.3 Mindfulness - Pairwise Comparisons

Comparison	Z	p-value (adj)	Mean Diff
< 1 Year - 1-5 Years	-0.76	1.00	-0.43
< 1 Year - 10+ Years	0.69	1.00	0.24
1-5 Years - 10+ Years	3.43	0.002**	0.67
< 1 Year - 5-10 Years	0.17	1.00	0.03
1-5 Years - 5-10 Years	1.74	0.24	0.46
10+ Years - 5-10 Years	-1.06	0.87	-0.21

2.2.4 Emotional Exhaustion - Pairwise Comparisons

Comparison	Z	p-value (adj)	Mean Diff
< 1 Year - 1-5 Years	-0.77	1.00	-0.47
< 1 Year - 10+ Years	0.68	1.00	0.32
1-5 Years - 10+ Years	3.47	0.002**	0.79
< 1 Year - 5-10 Years	0.21	1.00	0.03
1-5 Years - 5-10 Years	1.86	0.19	0.50
10+ Years - 5-10 Years	-0.97	1.00	-0.29

2.2.5 Burnout - Pairwise Comparisons

Comparison	Z	p-value (adj)	Mean Diff
< 1 Year - 1-5 Years	-1.73	0.25	-0.43
< 1 Year - 10+ Years	-0.17	1.00	-0.02
1-5 Years - 10+ Years	3.84	0.000***	0.41
< 1 Year - 5-10 Years	-0.53	1.00	-0.13
1-5 Years - 5-10 Years	2.26	0.07	0.30
10+ Years - 5-10 Years	-0.82	1.00	-0.11

3. Dropout Analysis

Comparison of baseline characteristics between completers (participants with all 3 timepoints) and dropouts.

Statistical tests used: Wilcoxon rank-sum test for continuous variables, Chi-square test for categorical variables.

Significance levels: * p < 0.05, ** p < 0.01, *** p < 0.001

3.1 Continuous Variables

Outcome (mean)	Completer	Dropout	Mean Difference	W	p-value
Age	47.03	44.24	2.80	12261.5	0.03*
Anxiety	1.60	1.76	-0.16	8259.5	0.29
Stress	2.42	2.50	-0.08	8367.5	0.52
Mindfulness	2.86	3.10	-0.25	8027.5	0.18
Emotional Exhaustion	3.63	3.85	-0.22	8344.5	0.30
Burnout	2.37	2.48	-0.11	8191.0	0.14
Depression	1.71	1.75	-0.04	8712.0	0.73
Recovery	4.46	4.35	0.11	9647.0	0.31
Job Crafting	5.03	4.94	0.08	9665.0	0.42
Ptsd	2.04	2.09	-0.04	8689.0	0.80
Social Support	4.19	4.28	-0.09	8603.5	0.28

3.2 Categorical Variables

Variable	χ^2	df	p-value
occupation	2.44	4	0.656
gender	1.38	2	0.501
tenure	7.74	3	0.052

3.3 Little's Test of Missing Completely At Random

χ^2	df	p-value
2.44	794	0.571

4. Piecewise growth model analyses with demographic control factors gender, age, occupation, tenure as co-variates

Model structure: outcome ~ time1 * time2 * component1 * component2 + occupation + gender + tenure + age + (1 | ID)

Significance levels: * p < 0.05, ** p < 0.01, *** p < 0.001

4.1 Models with Demands and Control

4.1.1 Social Support

Components: Demands × Control

Predictor	Estimate	SE	t-value	p-value
(Intercept)	4.033	0.331	12.17	< 0.001***
time1	0.331	0.107	3.09	0.002**
time2	-0.127	0.111	-1.14	0.255
Demands	-0.067	0.191	-0.35	0.728
Control	0.011	0.203	0.06	0.955
occupation	-0.020	0.046	-0.45	0.655
gender	0.036	0.100	0.36	0.720
tenure	0.043	0.091	0.48	0.633
age	0.005	0.007	0.72	0.472
time1:Demands	-0.441	0.147	-3.00	0.003**
time2:Demands	0.241	0.155	1.56	0.121
time1:Control	-0.288	0.156	-1.85	0.065
time2:Control	0.037	0.168	0.22	0.824
Demands:Control	0.002	0.280	0.01	0.993
time1:Demands:Control	0.690	0.215	3.22	0.001**
time2:Demands:Control	-0.299	0.230	-1.30	0.194

4.1.2 Job Crafting

Components: Demands × Control

Predictor	Estimate	SE	t-value	p-value
(Intercept)	4.007	0.241	16.65	< 0.001***
time1	0.059	0.088	0.67	0.504
time2	0.007	0.092	0.07	0.941
Demands	0.022	0.140	0.15	0.878
Control	-0.121	0.150	-0.81	0.420
occupation	0.046	0.033	1.41	0.159
gender	0.145	0.073	1.98	0.049*
tenure	0.011	0.065	0.17	0.861
age	0.015	0.005	3.09	0.002**
time1:Demands	-0.105	0.121	-0.87	0.385
time2:Demands	-0.081	0.130	-0.62	0.533
time1:Control	-0.168	0.128	-1.31	0.191
time2:Control	-0.160	0.141	-1.14	0.255
Demands:Control	-0.025	0.206	-0.12	0.903
time1:Demands:Control	0.300	0.178	1.68	0.093
time2:Demands:Control	0.277	0.193	1.43	0.153

4.1.3 Emotional Exhaustion

Components: Demands × Control

Predictor	Estimate	SE	t-value	p-value
(Intercept)	5.599	0.448	12.51	< 0.001***
time1	-0.070	0.119	-0.59	0.555
time2	-0.413	0.124	-3.35	< 0.001***
Demands	-0.653	0.245	-2.66	0.008**
Control	-0.381	0.260	-1.46	0.144
occupation	-0.080	0.061	-1.31	0.193
gender	0.198	0.134	1.48	0.140
tenure	0.065	0.122	0.53	0.596
age	-0.037	0.009	-3.94	< 0.001***
time1:Demands	-0.193	0.164	-1.18	0.238
time2:Demands	0.474	0.174	2.72	0.007**
time1:Control	0.012	0.173	0.07	0.944
time2:Control	0.574	0.187	3.06	0.002**
Demands:Control	0.524	0.358	1.46	0.144
time1:Demands:Control	0.222	0.241	0.92	0.357
time2:Demands:Control	-0.839	0.259	-3.25	0.001**

4.1.3 Burnout

Components: Demands × Control

Predictor	Estimate	SE	t-value	p-value
(Intercept)	3.476	0.205	16.96	< 0.001***
time1	0.006	0.061	0.10	0.922
time2	-0.245	0.063	-3.88	< 0.001***
Demands	-0.302	0.115	-2.63	0.009**
Control	-0.271	0.122	-2.22	0.027*
occupation	-0.047	0.028	-1.66	0.097
gender	0.047	0.062	0.75	0.452
tenure	0.036	0.056	0.63	0.527
age	-0.019	0.004	-4.53	< 0.001***
time1:Demands	-0.108	0.083	-1.30	0.194
time2:Demands	0.207	0.088	2.34	0.020*
time1:Control	-0.122	0.088	-1.38	0.167
time2:Control	0.323	0.095	3.40	< 0.001***
Demands:Control	0.317	0.168	1.89	0.060
time1:Demands:Control	0.229	0.122	1.88	0.060
time2:Demands:Control	-0.398	0.131	-3.04	0.003**

4.1.4 Model 5: Anxiety

Components: Demands × Control

Predictor	Estimate	SE	t-value	p-value
(Intercept)	2.645	0.179	14.74	< 0.001***
time1	0.037	0.053	0.70	0.485
time2	-0.268	0.056	-4.80	< 0.001***
Demands	-0.214	0.100	-2.14	0.033*
Control	-0.285	0.106	-2.69	0.007**
occupation	-0.046	0.024	-1.87	0.062
gender	0.053	0.054	0.98	0.326
tenure	0.029	0.049	0.60	0.551
age	-0.017	0.004	-4.49	< 0.001***
time1:Demands	-0.015	0.073	-0.21	0.833
time2:Demands	0.188	0.078	2.39	0.017*
time1:Control	-0.104	0.077	-1.34	0.181
time2:Control	0.282	0.085	3.34	< 0.001***
Demands:Control	0.202	0.146	1.38	0.168
time1:Demands:Control	0.033	0.107	0.31	0.756
time2:Demands:Control	-0.265	0.116	-2.28	0.023*

4.2 Models with Psychoeducation and Journaling

4.2.1 Social Support

Components: Psychoeducation × Journaling

Predictor	Estimate	SE	t-value	p-value
(Intercept)	4.092	0.324	12.64	< 0.001***
time1	0.134	0.112	1.19	0.235
time2	-0.010	0.122	-0.09	0.932
Psychoeducation	-0.430	0.198	-2.17	0.031*
Journaling	-0.216	0.198	-1.09	0.276
occupation	-0.004	0.045	-0.08	0.934
gender	0.044	0.099	0.44	0.660
tenure	0.052	0.090	0.58	0.566
age	0.005	0.007	0.71	0.481
time1:Psychoeducation	0.011	0.153	0.07	0.943
time2:Psychoeducation	-0.044	0.164	-0.27	0.790
time1:Journaling	-0.221	0.154	-1.44	0.152
time2:Journaling	-0.054	0.165	-0.33	0.743
Psychoeducation:Journaling	0.592	0.278	2.13	0.034*
time1:Psychoeducation:Journaling	0.431	0.214	2.01	0.045*
time2:Psychoeducation:Journaling	0.045	0.229	0.20	0.844

4.2.2 Mindfulness

Components: Psychoeducation × Journaling

Predictor	Estimate	SE	t-value	p-value
(Intercept)	4.215	0.366	11.51	< 0.001***
time1	0.335	0.136	2.46	0.014*
time2	-0.037	0.149	-0.25	0.802
Psychoeducation	0.430	0.221	1.94	0.053
Journaling	0.162	0.222	0.73	0.465
occupation	-0.043	0.050	-0.86	0.389
gender	0.149	0.111	1.35	0.179
tenure	-0.057	0.099	-0.57	0.569
age	-0.022	0.008	-2.85	0.005**
time1:Psychoeducation	0.070	0.183	0.38	0.703
time2:Psychoeducation	-0.298	0.197	-1.51	0.131
time1:Journaling	-0.315	0.183	-1.72	0.087
time2:Journaling	-0.048	0.198	-0.24	0.811
Psychoeducation:Journaling	-0.610	0.309	-1.98	0.049*
time1:Psychoeducation:Journaling	-0.156	0.254	-0.61	0.540
time2:Psychoeducation:Journaling	0.571	0.274	2.09	0.037*

4.3 Models with Engagement and Demands

4.3.1 Model 8: Mindfulness

Components: Engagement × Demands

Predictor	Estimate	SE	t-value	p-value
(Intercept)	4.496	0.373	12.06	< 0.001***
time1	0.314	0.130	2.42	0.016*
time2	-0.015	0.136	-0.11	0.912
Engagement	0.039	0.224	0.18	0.860
Demands	-0.464	0.215	-2.15	0.032*
occupation	-0.034	0.049	-0.68	0.498
gender	0.163	0.110	1.49	0.137
tenure	-0.034	0.098	-0.35	0.727
age	-0.022	0.007	-3.00	0.003**
time1:Engagement	-0.145	0.183	-0.79	0.429
time2:Engagement	-0.269	0.198	-1.36	0.175
time1:Demands	-0.155	0.181	-0.86	0.393
time2:Demands	-0.289	0.195	-1.48	0.138
Engagement:Demands	0.071	0.307	0.23	0.816
time1:Engagement:Demands	0.061	0.254	0.24	0.811
time2:Engagement:Demands	0.762	0.273	2.79	0.006**

Karolinska Institutet

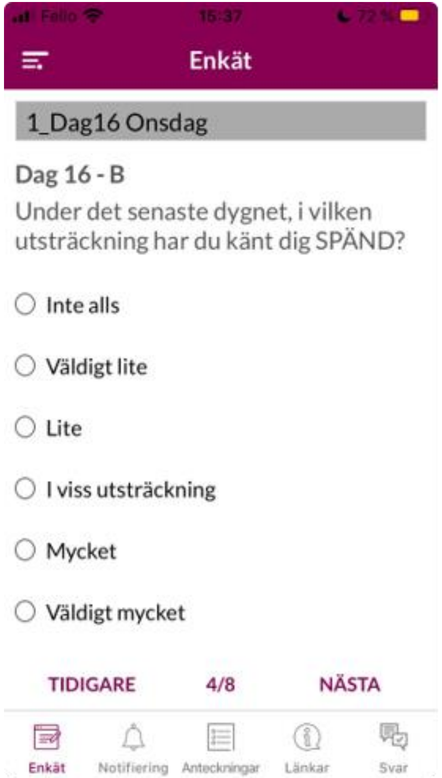

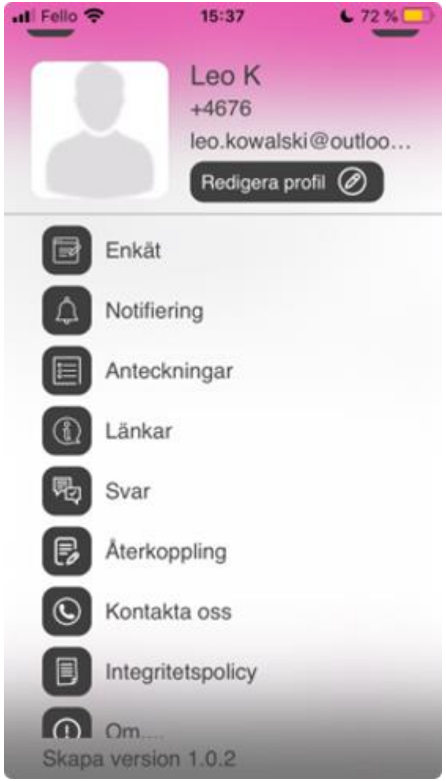

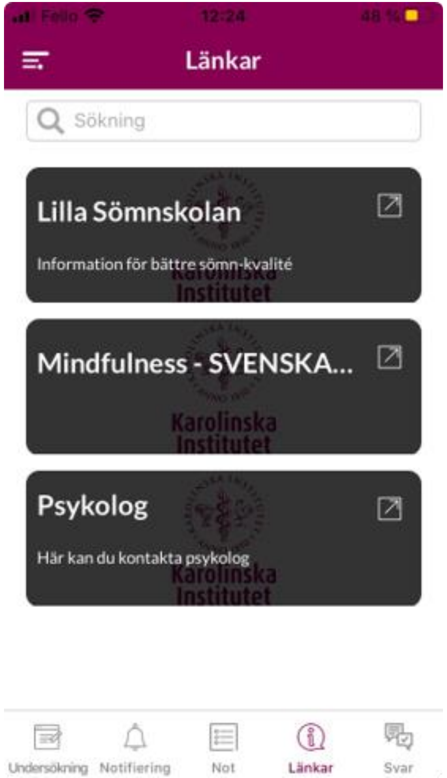

Supplement: Multimedia Appendix 1 [file formative-v9-e71032-s001.pdf]
